# Knowledge and attitudes of medical students in Lebanon toward artificial intelligence: A national survey study

**DOI:** 10.3389/frai.2022.1015418

**Published:** 2022-11-02

**Authors:** George Doumat, Darine Daher, Nadim-Nicolas Ghanem, Beatrice Khater

**Affiliations:** ^1^Faculty of Medicine, American University of Beirut, Beirut, Lebanon; ^2^Department of Family Medicine, Faculty of Medicine, American University of Beirut, Beirut, Lebanon

**Keywords:** artificial intelligence, medical education, medical student, choice of specialty, medical curriculum

## Abstract

**Purpose:**

This study assesses the knowledge and attitudes of medical students in Lebanon toward Artificial Intelligence (AI) in medical education. It also explores the students' perspectives regarding the role of AI in medical education as a subject in the curriculum and a teaching tool.

**Methods:**

This is a cross-sectional study using an online survey consisting of close-ended questions. The survey targets medical students at all medical levels across the 7 medical schools in Lebanon.

**Results:**

A total of 206 medical students responded. When assessing AI knowledge sources (81.1%) got their information from the media as compared to (9.7%) from medical school curriculum. However, Students who learned the basics of AI as part of the medical school curriculum were more knowledge about AI than their peers who did not. Students in their clinical years appear to be more knowledgeable about AI in medicine. The advancements in AI affected the choice of specialty of around a quarter of the students (26.8%). Finally, only a quarter of students (26.5%) want to be assessed by AI, even though the majority (57.7%) reported that assessment by AI is more objective.

**Conclusions:**

Education about AI should be incorporated in the medical school curriculum to improve the knowledge and attitudes of medical students. Improving AI knowledge in medical students will in turn increase acceptance of AI as a tool in medical education, thus unlocking its potential in revolutionizing medical education.

## Introduction

Artificial Intelligence (AI) is the ability of a computer to reach human-level performance in cognitive-based tasks (Turing, 1950). AI-powered technologies have been incorporated into finance, law, cybersecurity, manufacturing, computer science and other fields. Researchers have also been exploring the expansion of AI into medicine.

Modern medicine is rapidly evolving, and many fields have already integrated AI into clinical practice: in oncology for cancer diagnosis and grading (Londhe and Bhasin, 2019); in gastroenterology in context of endoscopes to detect and diagnose pathological lesions (Alagappan et al., 2018) and radiology to detect and interpret various cancerous entities on imaging (Hosny et al., 2018). AI has also found its way into medical education where it has been used in case-based E-learning (Khumrina et al., 2017) or history taking through virtual standardized patient systems (Maicher et al., 2019; Randhawa and Jackson, 2020). These tools can potentially revolutionize medical education, especially since ratings between the machine and three human raters were comparable in accuracy (Maicher et al., 2019).

It is generally accepted that AI will play an integral part in medicine, but its impact on medical students and their future remains unclear. Some studies show that it could steer people away from a career in medicine in general (Park et al., 2020) or from specialties considered more prone to be affected by AI like radiology (Pinto Dos Santos et al., 2019). Other studies show that students disagreed that physicians in general and radiologists would be replaced by AI (Pinto Dos Santos et al., 2019). One factor affecting the attitudes of medical students toward AI can be their knowledge about AI and its applications in medicine. The gap in knowledge stems from deficiencies in curriculum design to accommodate modern advances in medicine, such as AI. The failure of embedding AI material in the curriculum can be attributed to multiple factors. First, the lack of accreditation requirements pertaining to AI will leave the administrators without an incentive to expand their curriculum (Kolachalama and Garg, 2018). With calls for additional academic subjects and an ever-growing body of biomedical knowledge, medical schools are already having trouble keeping up their course offerings under the current framework. This problem is made worse by the fact that medical schools lack the faculty expertise needed to teach this material, which is mostly taught in faculties of computer science, mathematics, and engineering (Kolachalama and Garg, 2018).

Even though AI technology is rapidly gaining momentum in the medical field potentially revolutionizing medical education as a whole, the application of AI in medicine in Lebanon remains limited and restricted in some clinical and surgical fields, such as robotics (Labban et al., 2021). Similarly, the medical education curriculum offers limited educational content related to AI, which can lead to restricted knowledge and negative attitudes toward the topic. Several studies have assessed the knowledge and attitudes of medical students toward AI from all over the world, including the Middle East (Gong et al., 2019; Pinto Dos Santos et al., 2019; Sit et al., 2020; Ahmed et al., 2022; Al Saad et al., 2022). However, Research is still lacking regarding Lebanese medical students' perspectives and outlooks about AI. To the best of our knowledge, this is the first study exploring the medical students' knowledge and attitudes toward AI in medical education in Lebanon. Additionally, our study explores not only the effect of AI on the students' choice of specialty, but also on its potential on their educational journey. So, the purpose of this study is to shed light on these issues whilst assessing the students' baseline interest in learning more about the topic, as well as exploring the students' perspectives regarding the role of AI in medical education as both a subject in the curriculum and a teaching tool.

## Materials and methods

### Study design

This is a cross-sectional study aiming to assess the knowledge, attitudes, and readiness to learn about Artificial Intelligence, among medical students in Lebanon using a self-administered online questionnaire with close-ended questions using Limesurvey. The Institutional Review Board (IRB) at AUB approved of this study. The questions within the survey were used to construct knowledge and attitude scores. A knowledge score was constructed out of 15 knowledge-related questions and an attitude score was constructed out of the 5 attitude-related questions.

### Sampling strategy

Our study population includes medical students in the 7 medical schools in Lebanon at all academic levels. Recruitment of AUB students was done *via* email address, using IRB-secured mailing list. For the rest, the snowballing method was used *via* social media (Whatsapp, Instagram, LinkedIn, Twitter) to share the Limesurvey link. Class representatives (identified *via* the Lebanese Medical Students' International Committee (LEMSIC) and other Lebanese medical students' social media groups) were contacted and asked to share the survey link to their respective class group. The survey invitation was sent in September 2021 and remained open for 6 weeks. Two reminder emails were sent 2 weeks apart.

### Inclusion criteria

This study includes medical students in the 7 Lebanese medical schools, at all medical levels.

### Exclusion criteria

Students who did not receive the link to the survey and those who did not sign the consent form.

### Data collection and analysis

Data was collected through an anonymous self-administered survey available in English *via* the LimeSurvey platform hosted by the AUB server. The survey link reached the population *via* email or social media. Only participants who agree to the informed consent form on the survey's landing page were eligible to participate. Data analysis was done using IBM SPSS version 24. Participants who filled more than 75% of the survey were included. 75% completion of this survey translates to completion of all the sections except the fourth section; the medical education part. As such, those that completed the first three sections were analyzed separately from those who completed the entire survey. Likert scale questions that included 4 possibilities were transformed into two by merging the “strongly agree” and “agree” options, in addition to the “strongly disagree” and “disagree” options. Knowledge and attitudes scores were computed by assigning a value of 1 to each correct answer and 0 to each incorrect one. Chi-square and one-way analysis of variance (ANOVA) tests were used to determine the association between the independent categorical variables. Logistic regression was used to predict the likelihood of the relationship between our dependent and independent variables. Significance level was set as *p*-value of equal to or less than 0.05.

## Results

### Demographic characteristic of the participants

Out of a total of 3,050 medical students in Lebanon, 294 (9.6%) filled the survey, 206 (6.8%) filled at least 75% of it, and, of those, 201 (6.6%) students filled it completely. Gender distribution was 119 (57.8%) males and 87 (42.2%) females, with a mean age of 22.7. When assessing the students' source of AI knowledge, 31 (15%) students acquired their AI knowledge from a university level course, 20 (9.7%) from medical school curriculum, 23 (11.2%) from medical research project, 167 (81.1%) from media, and 43 (16%) from other sources. Participants' undergraduate majors and academic level were assessed as shown in Table 1. The mean knowledge score was 7.79 ± 2.78 (out of 15) and the mean attitude score was 3 ± 0.94 (out of 5).

**Table 1:** Demographic characteristics of the participants and differences in knowledge and attitude scores.

	**N/%**	**Knowledge score (n/15)**	**Attitude score (n/5)**
**Gender**	206		
Female	87 (42.2%)	7.37 + 2.70	2.92 ± 0.92
Male	119 (57.8%)	8.09 ± 2.79	3.06 ± 0.96
*P*-value		0.064	0.296
**Academic level**	206	
Clinical	124 (60.2%)	8.18 ± 2.84	2.99 ± 0.92
Preclinical	82 (39.8%)	7.20 ± 2.58	3.01 ± 0.98
*P*-value		**0.013**	0.880
**Undergraduate major**	206		
Biology	132 (64.1%)	7.98 ± 2.66	2.95 ± 0.93
Chemistry	21 (10.2%)	7.24 ±3.24	2.81 ±1.17
Physics	6 (2.9%)	9.17 ± 4.12	3.5 ± 0.55
Social science	12 (5.8%)	8.25 ± 2.38	2.92 ± 0.52
Other	35 (17%)	7.00 ± 2.69	3.23 ± 0.97
*P*-value		0.194	0.288
**Source of knowledge**	206		
University level course			
Yes	175 (85%)	8.94 ± 3.11	3.02 ± 0.95
No	31 (15%)	7.58 ± 2.67	2.87 ± 0.89
*P*-value		**0.012**	0.409
Medical school curriculum			
Yes	20 (9.7%)	9.55 ± 2.21	3.02 ± 0.96
No	186 (90.3%)	7.60 ± 2.77	2.80 ± 0.77
*P*-value		**0.003**	0.319
Medical research project			
Yes	23 (11.2%)	9.78 ± 2.54	3.30 ± 0.88
No	183 (88.8%)	7.54 ± 2.71	2.96 ± 0.95
*P*-value		**< 0.001**	0.100
Media			
Yes	167 (81.1%)	7.81 ± 2.87	3.02 ± 0.93
No	39 (18.9%)	7.69 ± 2.38	2.90 ± 1.00
*P*-value		0.815	0.452
Other (206)			
Yes (33)	33 (16%)	8.18 ± 2.64	2.91 ± 1.01
No (173)	173 (84%)	7.71 ± 2.80	3.02 ± 0.93
*P*-value		0.373	0.547

Bold values represent significant results (*p* < 0.05).

### Knowledge and attitude

Students at the clinical level had significantly higher knowledge scores (8.18 ± 2.84) as compared to their peers at the preclinical level (7.20 ± 2.58). However, no statistically significant change in attitude score was noted between the two academic levels. When comparing scores based on source of knowledge, students who acquired their knowledge through a university level course had statistically significant higher knowledge scores (8.94 ± 3.11 vs. 7.58 ± 2.67, *p*-value = 0.012). Similarly, students who received their knowledge from medical school curriculum had a statistically significant higher knowledge scores (9.55 ± 2.21 vs. 7.60 ± 2.77, *p*-value = 0.03) but no difference in attitude scores. Furthermore, knowledge scores of students who selected “medical research project” as a source of AI knowledge were statistically higher than those who did not select it (9.78 ± 2.54 vs. 7.54 ± 2.71). However, there was no statistically significant difference in attitude scores to be noted between the students based on knowledge source.

### Choice of specialty

**Figure 1:**
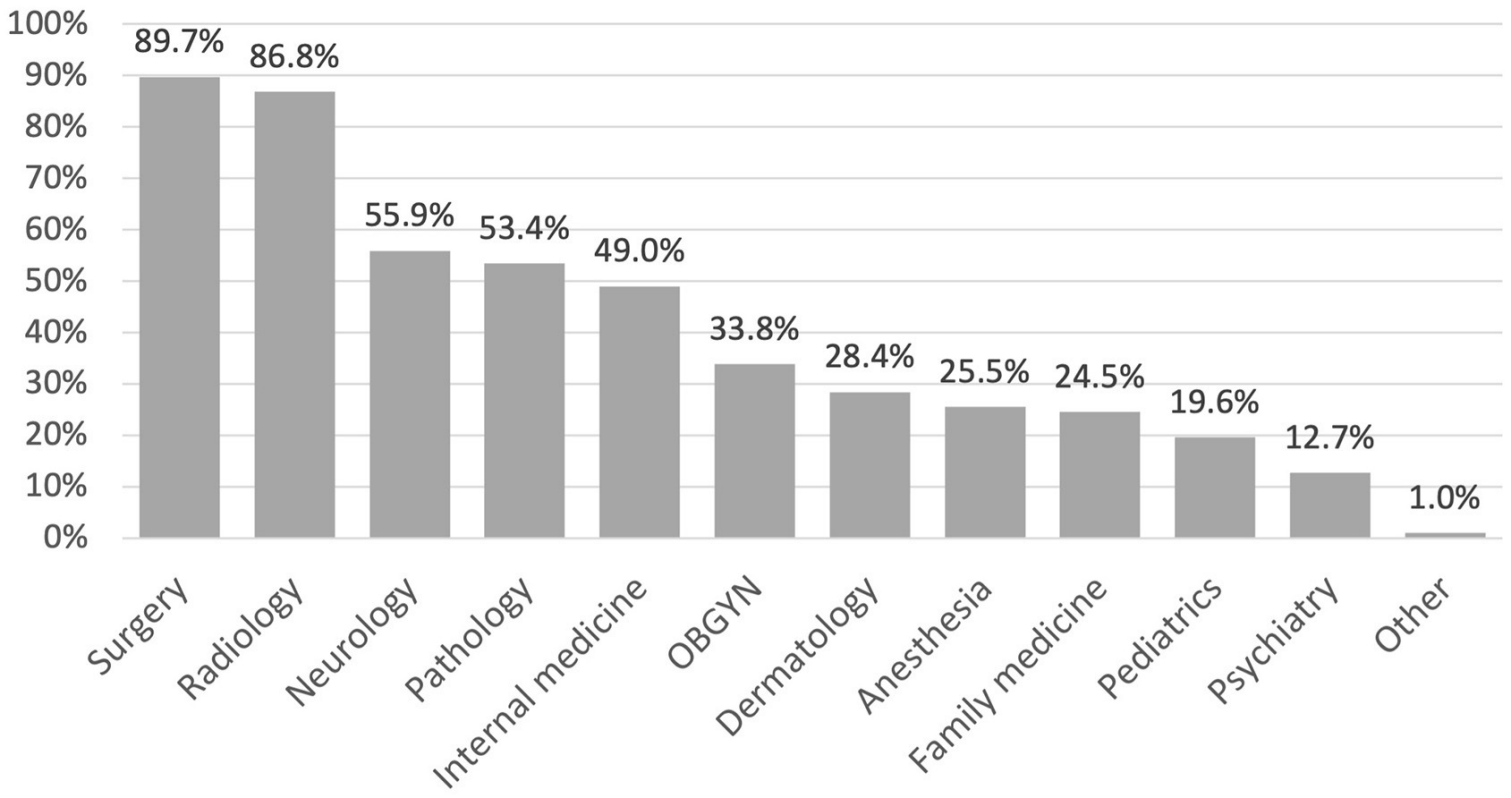
The current use of AI by medical specialty as perceived by medical students.

Participants were asked to select medical specialties that incorporate AI. The highest selected specialties were surgery (89.7%) and radiology (86.8%) whereas pediatrics (19.6%) and psychiatry (12.7%) were selected the least (Figure 1).

Table 2 is a crosstab between students' choice of specialty and their belief on the effect of AI on their choice. All of the students who chose pathology as their specialty of choice (100%) and most of those who chose radiology (77.8%) answered “yes” when asked if AI development is affecting their choice. On the other hand, students who selected psychiatry (85.7%) and family medicine (80%) have the highest “no” responses when asked if AI development is affecting their choice.

**Table 2:** Choice of specialty by the effect of AI advancement on the choice.

**Choice of**	**Is the development of AI in medicine**	
**specialty**	**affecting your choice of specialty?**	
	**Yes = 56**	**No = 153**
	**(26.8%)**	**(73.2%)**
Surgery (n = 76)	22 (28.9%)	54 (71.1%)
Internal medicine (n = 77)	16 (20.8%)	61 (79.2%)
Family medicine (n = 10)	2 (20%)	8 (80%)
Radiology (n = 9)	7 (77.8%)	2 (22.2%)
Psychiatry (n = 14)	2 (14.3%)	12 (85.7%)
OBGYN (n = 22)	6 (27.3%)	16 (72.7%)
Pediatrics (n = 21)	6 (28.6%)	15 (71.4%)
Neurology (n = 18)	4 (22.2%)	14 (77.8%)
Dermatology (n = 13)	3 (23.1%)	10 (76.9%)
Pathology (n = 3)	3 (100%)	0 (0%)
Anesthesia (n = 8)	4 (50%)	4 (50%)
Ophthalmology (n = 4)	2 (50%)	2 (50%)
Emergency medicine (n = 3)	1 (33.3%)	2 (67.7%)
Radiation oncology (n = 1)	0 (0%)	1 (100%)
I don't know (n = 47)	15 (31.9%)	32 (68.1%)

Table 3 further explores the differences between participants that believe that advancement in AI is affecting their choice of specialty and those who do not, based on demographic characteristics using Chi-squared and ANOVA tests. The only significant difference is in the knowledge score, where participants who believe that AI is affecting their choice of specialty (8.59 ± 2.76) have higher scores than those who do not (7.50 ± 2.73).

**Table 3:** Differences in the effect of AI on choice of specialty based on demographics.

**Characteristic**	**The development of AI**		
**(n = 206)**	**affecting the choice of specialty**		
	**Yes = 54**	**No = 152**	***P*-**
	**(26.2%)**	**(73.8%)**	**value**
**Gender**
Female (87)	20	67	0.368*
Male (119)	34	85	
**Academic level**			
Clinical (124)	29	95	0.257*
Preclinical (82)	25	57	
**Undergraduate major**
Biology (132)	29	103	0.417*
Chemistry (21)	6	15	
Physics (6)	2	4	
Social science (12)	4	8	
Other (35)	13	22	
**Score**
Knowledge	8.59 ± 2.76	7.50 ± 2.73	0.013**
Attitude	2.87 ± 0.91	3.05 ± 0.08	0.240**

^*^Chi-squared test.

^**^ANOVA.

### Medical education

Students were asked about their attitudes toward AI in medical education. The questions explored whether they consider that assessment would be more objective, if the feedback they would get would be more personalized, and if they would prefer learning from personalized AI-generated questions (Table 4). ANOVA was used to compare answers with knowledge and attitude scores. There were no statistically significant differences in scores between the two answer groups.

**Table 4:** Frequency table of the answers to the survey's questions.

**Question**	**Agree % (n)**	**Disagree % (n)**
Knowledge (n = 206)
I have a good understanding of the basics of artificial intelligence	59.7% (123)	40.3% (83)
Deep learning algorithms can “learn” by supplying it with data, without the help of human engineers	55.8% (115)	44.2% (91)
For AI to “learn”, it requires a large amount of labeled data (information already processed by a human and clearly labeled)	83.5% (172)	16.5% (34)
The current AI programs are good at pattern recognition but not deduction	67% (138)	33% (68)
I understand the limitations of AI in medicine	74.8% (154)	25.2% (52)
Attitude (n = 206)
AI will play an important role in healthcare	95.6% (197)	4.4% (9)
Some human specialists will be replaced by AI in the near future	55.8% (115)	44.2% (91)
AI will never render human-doctors expendable	79.1% (163)	20.9% (43)
Some specialties are more prone to be replaced by AI than others	90.3% (186)	9.7% (20)
The developments of AI make medicine more interesting to me	71.4% (147)	28.6% (59)
Medical Education (n = 201)		
Would you feel more objectively assessed and graded if an AI system graded you during the clinical skills exam instead of the usual standardized preceptor?	57.7% (116)	42.3% (85)
If you were to be assessed by an AI system during your clinical skills exam, do you feel that you would receive more personalized feedback than with a human standardized patient?	34.3% (69)	65.7% (132)
Would you prefer learning from personalized AI generated questions as opposed to traditional questions (from textbooks, question banks, etc...)?	54.7% (110)	45.3% (91)

Chi-squared and ANOVA tests were used to compare the differences based on demographics between the students who would like to be assessed by an AI and those who do not or do not know. The only statistically significant difference between the three answer groups was in attitude scores. A Tukey *post-hoc* test revealed that the attitude score was statistically significantly higher in the group that answered “yes” (3.28 ± 0.82, *p* = 0.015) compared to the group that answered no (2.81 ± 0.98). There was no statistically significant difference between the groups that answered, “yes” and “I don't know” (*p* = 0.251) or the groups that answered “no” and “I don't know” (*p* = 0.390).

To further explore the question, a multinomial logistic regression was performed to create a model of the relationship between the knowledge and attitude scores and the willingness to be assessed by AI (“yes”, “no”, “I don't know”). The fit between the model containing only the intercept and data improved with the addition of the predictor variables, X^2^(4, *N* = 200) = 14.477, *p* = 0.006. The model predicted that it is more likely to answer “yes” than “I don't know” to whether they want to be assessed by AI if your knowledge score is higher.

Moreover, when asked about their preferred method to learn suturing, 27.9% of the participants preferred only human surgeons as teachers, 1.5% chose AI only, and 70.6% chose a combination of AI and human surgeons.

Finally, answers to the survey's questions can be found reported as frequencies in Table 4.

## Discussion

### Knowledge

In this online survey of Lebanese medical students, we found that around 60% (59.7%) believe they have a good understanding_of AI basics, which contrasts with the 30.8% demonstrated in a multicentric German study by Santos et al. (Pinto Dos Santos et al., 2019) and 44.6% in a multicentric UK study by Sit et al. (Sit et al., 2020). Furthermore, there was no statistically significant difference in terms of knowledge when comparing male and female respondents in our study where 23% of females vs. 28.6% of males chose yes (*p* = 0.368). This contrasts with the study by Santos et al. which showed a statistically significant difference when comparing male and female respondents, with males more likely to answer “yes” (Pinto Dos Santos et al., 2019).

With regards to sources of AI knowledge, only 9.7% of Lebanese medical students reported receiving their knowledge from the medical school curriculum as opposed to 55.9% in the German study (Pinto Dos Santos et al., 2019), and 0% in the UK study by Sit et al. (Sit et al., 2020). 81.1% of our respondents reported receiving their knowledge from media, entailing both mass media and social media, which is comparable to the 85.2% reporting receiving AI information from media and 65.8% from social media, in the German study by Pinto Dos Santos et al. (2019). Despite that a substantial proportion of medical students in Lebanon receive their knowledge about AI from media, our study showed that those who received their knowledge from media did not have statistically significant higher knowledge scores from those who got their knowledge from other sources (*p*-value = 0.815). However, students who received their knowledge about AI from medical school curricula (9.55 ± 2.21, *p*-value = 0.003), medical school research projects (9.78 ± 2.54, *p*-value < 0.001) and undergraduate courses (8.94 ± 3.11, *p*-value = 0.012) had statistically significantly higher knowledge scores than their peers who did not. Furthermore, students in their clinical years had statistically significantly higher knowledge scores than their peers in preclinical years (*p*-value = 0.013) which might be explained by more real-life exposure to the usages of AI in clinical practice. These results illustrate the importance of incorporating AI into the medical curriculum in a structured and well-thought-out manner. Better quality sources for AI education need to be adopted and implemented within the formal curriculum to improve knowledge on the topic.

With regards to the current limitations of AI, 74.8% of our respondents reported understanding them, which is higher than 48.3%, the percentage of respondents in the Sit et al. study that selected strongly agree or agree to this question (Sit et al., 2020).

Even though our respondents may be more exposed to AI teaching through formal channels i.e., medical school curricula than their peers in the UK, more work needs to be done to standardize and disseminate AI knowledge within Lebanese medical school curricula since 9.7% can still be considered a low number.

### Attitudes

In our study, students who had formal instruction on AI, i.e., from medical school curricula, research projects and undergraduate courses, did not have a statistically significant difference in the attitude score compared to the students who did not have a formal training. Even though the formally trained students had statistically significant higher knowledge scores, as discussed before, and this might illustrate the fact that it is not enough to merely incorporate AI into medical school curricula. Further studies to explore factors that might influence medical students' attitude toward AI are needed.

Most of our respondents (95.6%) believe that AI will play an important role in healthcare, with no statistically significant difference in attitude scores between those in their clinical and pre-clinical years. Similarly, in a US study by Park et al., over 75% of students who are members of radiology interest groups believe that AI would have a moderate-to-major effect on medicine during their careers (Park et al., 2020). In the same study, first-year medical students were more likely to believe that AI will have a profound impact on medicine than fourth-year medical students (M1 = 82%, M4 = 65%). This is in contrast to our study as there was no statistically significant difference in attitude scores between clinical and pre-clinical students (Park et al., 2020).

55.8% of our respondents agreed that some human specialists will be replaced by AI in the near future, which is in stark difference to results by the study of dos Santos et al. which showed that 96.6% of students disagreed with the statement that human physicians in general could be replaced in the foreseeable future (Pinto Dos Santos et al., 2019). 71.4% of our respondents report that advancements in AI make medicine more exciting as compared to 44.5% of students in the German study (Pinto Dos Santos et al., 2019).

Most of our respondents (90.3%,) agreed that AI will affect certain medical specialties more than others in terms of specialists being prone to be replaced by AI and job reduction. Similar results were found amongst the respondents in the provincial study by Mehta et al. (2021) (250/288, 87%). We need to further evaluate the main specialties respondents think will be negatively impacted by AI in terms of job opportunities in hopes of uncovering misconceptions, if any, that can be addressed. Subsequently, medical students will be able to make informed choices about the specialties they want to delve into without factoring in potentially unfounded concerns.

### Choice of specialty

Our survey showed that 26.2% of respondents agreed that development of AI is affecting their choice of specialty. This result is very similar to the results obtained by Mehta et al. (2021) (25%) but lower than the results of the study by Park et al. (2020) (44%). Moreover, our results demonstrated that the mean knowledge score of students whose choice of specialty is impacted by AI is higher as compared to those whose choice of specialty is not impacted by AI. This reinforces the need for adequate and trustworthy knowledge sources about AI.

Another way to approach the effect of AI on students' choices is by trying to elucidate which fields they felt were more affected. To do so, we categorized students by their choice of specialty and subsequently assessed whether they felt that AI affected their choice of specialty. The majority of those who chose radiology and pathology believe that AI is affecting their choice of training. As compared to radiation oncology and psychiatry, where only a minority believed that their choice of specialty was affected by the development of artificial intelligence. Similar results affecting specific fields are evident in the literature, for example, a survey conducted by Park et al. in 2021 showed that students believe that diagnostic radiology and surgery would be impacted the earliest and to the most extent by AI (Park et al., 2020).

This highlights the need to go beyond merely providing general education about AI in medicine. Education about AI ought to be tailored to include the different features and characteristics of different specialties to eliminate the discrepancies in understanding that may arise.

### Medical education

The versatility and vast potential of AI allows it to serve as a tool in the teaching and assessment of medical students. One specific area where AI could be incorporated is in the Objective Structured Clinical Examination (OSCE). Since the OSCE is a knowledge-based exam, AI can help students prepare for exams by creating standardized patients from data modeled based on real patients' charts (Soong and Ho, 2021). AI could also act as an objective evaluator with the advantage of a fast and real-time feedback. Even though 57% of medical students in our study felt that an assessment by AI would be more objective than that of a human examiner, only 26.4% of them said they agree to be evaluated by AI. Furthermore, only 34.3% believe that AI can provide them with immediate feedback after the exam. One explanation for students' hesitancy regarding the adoption of AI evaluators in OSCE examinations could be a lack of knowledge on AI features like immediate and objective feedback. Adequate exposure of students to the applicability of AI as an evaluation and teaching tool can potentially alter their misconceptions on the limitations of the algorithms. Subsequently, students will be equipped with an objective knowledge base to form an informed opinion about whether to accept or reject the role of AI in evaluating them. This is supported by our logistic regression which demonstrated that a student is more likely to answer “yes” when asked if they want to be assessed by AI if they had higher knowledge or high attitude scores.

Our respondents were more inclined to accept the technology as a compliment to the human aspect of teaching rather than replacing the current teaching tools, with 54.7% of respondents preferring to learn from personalized AI-generated questions compared to traditional practice questions, and 70.6% preferring to learn suturing from a combination of AI and human surgeons.

Finally, most respondents (88.56%) were interested in attending a workshop about AI in medicine. Students are enthusiastic to explore the field, and formally introducing AI into the curriculum can serve as a steppingstone to start from and build upon.

### Strengths and limitations

The study addresses an important yet unexplored issue among Lebanese medical students and helps to better understand the problem in hopes of devising solutions. To date, this is the first study that assessed the knowledge and attitudes toward AI in medical education in an Arab country. Some limitations include the low response rate (around 6.8%) which can affect generalizability and representation. Another possible limitation is the sampling strategy since using social media and snowballing limits randomization and thus generalizability.

## Conclusion

This survey explored the knowledge and attitudes of medical students in Lebanon toward AI and found overall enthusiasm about the topic. With the explosion of AI into every medical domain, the incorporation of AI-related education into medical school curricula becomes paramount. Further studies and targeted focus groups should be undertaken to elucidate what exactly needs to be tackled to address the gaps of knowledge and hesitancy in attitudes. The place and use of AI-related technologies in medical education as assessment and education tools for students still needs to be explored. It would also be interesting to explore the attitude of medical educators toward AI and its role in medical education.

## Data availability statement

The raw data supporting the conclusions of this article will be made available by the authors, without undue reservation.

## Ethics statement

The studies involving human participants were reviewed and approved by the Institutional Review Board (IRB) at the American University of Beirut (AUB). The patients/participants provided their written informed consent to participate in this study.

## Author contributions

GD and DD contributed equally to this article, did literature review, collected the data, prepared the tables, and wrote the first draft of manuscript. GD conceived the idea of this study and analyzed the data. N-NG and BK helped with writing, reviewing, and editing the manuscript. BK was responsible for the supervision of this project. All authors approved the final version of this article.

## Conflict of interest

The authors declare that the research was conducted in the absence of any commercial or financial relationships that could be construed as a potential conflict of interest.

## Publisher's note

All claims expressed in this article are solely those of the authors and do not necessarily represent those of their affiliated organizations, or those of the publisher, the editors and the reviewers. Any product that may be evaluated in this article, or claim that may be made by its manufacturer, is not guaranteed or endorsed by the publisher.

## Abbreviations

AI: artificial intelligence
OSCE: objective structured clinical examination.
